# Functional Disability, Violence, HIV Status, and Risk Factors for HIV Among Adolescent Girls and Young Women — Eswatini, 2022

**DOI:** 10.15585/mmwr.mm7409a2

**Published:** 2025-03-20

**Authors:** Ghenet Besera, Francis B. Annor, Elizabeth A. Swedo, Laura F. Chiang, Sana N. Charania, Phumzile Mndzebele, Michelle J. Li, Jennifer Hegle, Anne Laterra, Robyn A. Cree, Nozipho Nzuza-Motsa, Thobile Mkhonta, Kristopher Mills, Silke Felton, Greta M. Massetti

**Affiliations:** ^1^Epidemic Intelligence Service, CDC; ^2^Division of Violence Prevention, National Center for Injury Prevention and Control, CDC; ^3^Division of Global HIV and TB, Global Health Center, CDC Eswatini; ^4^Division of Global HIV and TB, Global Health Center, CDC; ^5^Division of Human Development and Disability, National Center on Birth Defects and Developmental Disabilities, CDC; ^6^Ministry of Health, Government of Eswatini, Mbabane, Eswatini; ^7^U.S. Agency for International Development, Mbabane, Eswatini; ^8^U.S. Agency for International Development, Washington, DC; ^9^U.S. Agency for International Development, Pretoria, South Africa; ^10^Office of the Director, National Center for Injury Prevention and Control, CDC.

**Summary**

**What is already known about this topic?**

Eswatini has made substantial progress in addressing its HIV epidemic and violence against children. However, adolescent girls and young women, particularly those with disabilities, might remain at risk for HIV infection and for experiencing violence.

**What is added by this report?**

In Eswatini, adolescent girls and young women aged 13–24 years with functional disabilities (difficulties in performing activities) had higher prevalences of experiencing sexual, physical, and emotional violence, but in adjusted analyses, disability was not associated with higher prevalence of HIV infection.

**What are the implications for public health practice?**

Collaboration between disability-serving organizations and violence prevention partners across health, education, and social welfare sectors might help reach priority populations and provide disability-inclusive violence prevention programming.

## Abstract

Eswatini has made substantial progress responding to its HIV epidemic and reducing violence against children. However, adolescent girls and young women with disabilities might be at increased risk for experiencing violence and for HIV infection, compared with those without disabilities. Data from the 2022 Eswatini Violence Against Children and Youth Survey were analyzed to compare HIV infection and violence-related measures by functional disability status (e.g., difficulties in performing functional activities such as seeing, walking, or communicating) among adolescent girls and young women. In 2022, in Eswatini, 14.0% of adolescent girls and young women aged 13–24 years had a reported functional disability. Compared with those without a functional disability, adolescent girls and young women with a functional disability had higher lifetime prevalences of experiencing sexual, physical, and emotional violence. They were also more likely to know where to seek help for experiences of violence. After adjusting for sociodemographic characteristics, HIV testing and infection status, HIV risk factors, sexual risk behaviors, and HIV treatment and prevention services did not differ by functional disability status. Prioritizing accessible, disability-inclusive prevention programs and services might help reduce experiences of violence among adolescent girls and young women with disabilities. Partnering with disability-led and disability-serving organizations and directly with adolescent girls and young women with disabilities to plan and implement programs and services that are disability-inclusive could help ensure that adolescent girls and young women with disabilities are aware of and can access these resources.

## Introduction

Eswatini has made substantial progress in HIV epidemic control and in reducing violence against children (*1*,*2*). However, certain populations remain particularly vulnerable to HIV infection and violence. Adolescent girls and young women in Eswatini are disproportionately affected by HIV, with an estimated prevalence of HIV infection among those aged 15–24 years (11.0%) that is more than three times that among male peers (3.4%) (*2*). Adolescent girls and young women with disabilities are particularly at risk for violence and HIV infection because of physical or communication barriers to accessing HIV prevention, testing, and treatment services, in addition to economic vulnerabilities, exclusion from education, and discrimination (*3*–*5*). This report describes self-reported functional disability prevalence (difficulties in performing functional activities [e.g., seeing, walking, or communicating]) (*6*) and the association with HIV and violence-related measures among adolescent girls and young women aged 13–24 in Eswatini. Findings could be used to improve service delivery and better understand the risks and needs of adolescent girls and young women with disabilities.

## Methods

### Data Source

Data from 6,318 adolescent girls and young women aged 13–24 years who participated in the 2022 Eswatini Violence Against Children and Youth Survey (VACS) were analyzed (female response rate = 90.1%). VACS is a cross-sectional, nationally representative household survey of persons aged 13–24 years that collects data on experiencing violence, HIV infection, and risk and protective factors for violence and HIV infection (*1*). Participation in VACS is voluntary, and for participants aged 13–17 years, parental permission and assent from the participant are obtained; for those aged ≥18 years, participant consent is obtained. Sex-matched interviewers conduct the interviews and record responses electronically using tablets. Forty-nine persons with severe disabilities or challenges in understanding or responding to questions were excluded. A comprehensive response plan and referral protocol was in place for participants who needed referrals during or after the survey, including those who recently experienced violence (*1*).

### Disability Measures

Functional disability status was assessed using a modified version of the Washington Group on Disability Statistics Short Set (WG-SS) on Functioning questionnaire (*6*) (Supplementary Table, https://stacks.cdc.gov/view/cdc/176833#tabs-3). This analysis considered adolescent girls and young women to have a disability if they responded, “some difficulty,” “a lot of difficulty,” or “cannot do at all” to at least one question assessing six domains of current functioning: vision, cognition, mobility, self-care, independent living,* and communication.^†^ Because the ability to hear an interviewer is required for participation in an interviewer-administered survey, the WG-SS question on hearing was not included in the Eswatini VACS.

### Violence-Related Measures

Violence-related measures included lifetime experiences of sexual, physical, and emotional violence; knowledge of a place to go for help for experiences of violence and to seek help for sexual and physical violence; having sought professional services for sexual and physical violence; and having received professional services for experiences of sexual and physical violence.

### HIV Testing, Prevention, and Treatment Measures

HIV testing and infection status measures included ever having been tested for HIV infection, tested positive for HIV, and knowing one’s HIV infection status. HIV status was ascertained either via self-report of a previous positive HIV test result or positive rapid HIV test result at the time of the VACS interview. HIV testing was offered using the national HIV rapid testing algorithm (*1*). HIV prevention measures and treatment included knowledge of and ever having taken pre-exposure or postexposure prophylaxis, and among adolescent girls and young women living with HIV, being on antiretroviral therapy and viral load suppression. 

### HIV Infection Risk Factors and Sexual Risk Behaviors

HIV risk factors and sexual risk behaviors included lifetime experience of transactional sex, ever having symptoms or received a diagnosis of a sexually transmitted infection, forced sexual initiation, early sexual debut; and any of the following during the previous 12 months: multiple sexual partners, infrequent condom use (sometimes or never using condoms), positive or unknown HIV status of sex partners, sex partners who were ≥5 years older than the respondent, partners who ever refused to wear a condom, and fear of experiencing violence from disclosure of HIV status if the respondent received a positive HIV test result. Because of complex skip patterns used in VACS (i.e., each respondent could receive a different sequence of questions based on prior responses), indicator denominators might differ. All measures were dichotomized (i.e., yes or no) and self-reported during face-to-face interviews.

### Data Analysis

Prevalence estimates were calculated for number of functional disabilities, functional disability status, and sociodemographic characteristics and HIV and violence-related measures by functional disability status. Rao-Scott chi-square tests were used to assess differences in sociodemographic characteristics by functional disability status, with p-values <0.05 considered statistically significant. Associations between functional disability status (independent variable) and HIV and violence-related measures (dependent variables) were assessed in separate unadjusted and adjusted logistic regression models, which generated prevalence ratios (PRs) comparing HIV and violence-related measures by disability status.^§^ To adjust for potential confounders of the association between functional disability and the different measures, adjusted analyses controlled for sociodemographic variables that reflect potential social and environmental influences (age, education, food insecurity,^¶^ orphan status,** marital status, and residence^††^). To account for multiple statistical tests, a Bonferroni-corrected significance level of p<0.0017 was used for regression analyses. Survey weights were included for all analyses. Analyses were conducted using SAS (version 9.4; SAS Institute), accounting for the complex survey design. This activity was reviewed by CDC, deemed not research, and was conducted consistent with applicable federal law and CDC policy.^§§^

## Results

### Functional Disability Prevalence

Among adolescent girls and young women aged 13–24 years in Eswatini, 14% have a self-reported functional disability, with 11.2% reporting one functional disability type and 2.8% reporting two or more types (Table 1). The most commonly reported functional disability domain was vision (6.7%).

**TABLE 1:** Prevalence of functional disability among adolescent girls and young women aged 13–24 years, by disability domain (N = 6,318) — Violence Against Children and Youth Survey, Eswatini, 2022

Functional disability	No., unweighted	Weighted % (95% CI)
Functional disability domain*		
Vision	404	6.7 (5.7–7.7)
Cognition	181	3.0 (2.3–3.7)
Mobility	161	2.9 (2.2–3.6)
Self-care	60	0.9 (0.6–1.1)
Independent living^†^	86	1.5 (1.1–1.9)
Communication	180	2.8 (2.2–3.5)
**Number of functional disabilities**		
None	5,465	86.0 (84.5–87.5)
One	690	11.2 (10.0–12.4)
Two or more	163	2.8 (2.2–3.5)
**Functional disability status**		
Functional disability in at least one functional domain	853	14.0 (12.5–15.5)
No functional disability in any domain	5,465	86.0 (84.5–87.5)

* Self-reported, “some difficulty,” “a lot of difficulty,” or “cannot do at all” to one or more functional disability domains (vision, cognition, mobility, self-care, independent living, or communication). Because the ability to hear an interviewer is required for participation in an interviewer-administered survey, the Washington Group Short Set on Functioning question on hearing was not included in the Eswatini Violence Against Children and Youth Survey.

^†^ Independent living is not included in the Washington Group on Disability Statistics Short Set on Functioning questionnaire. A modified version (for country-specific daily errands) of the American Community Survey question on independent living was included in the Eswatini Violence Against Children and Youth Survey. https://www.census.gov/topics/health/disability/guidance/data-collection-acs.html

### Associations of Violence and HIV Measures with Functional Disability

No differences were observed in characteristics between adolescent girls and young women aged 13–24 years with versus without functional disability except more of those with a functional disability experienced food insecurity (67.0% versus 59.7%; p = 0.002) (Table 2). After adjusting for sociodemographic characteristics, adolescent girls and young women with a functional disability had a higher prevalence of experiencing lifetime sexual violence (adjusted prevalence ratio [aPR] = 2.0), physical violence (aPR = 1.7), and emotional violence (aPR = 2.2) versus those without a functional disability (Table 3). Adolescent girls and young women with versus without a functional disability were more likely to know of a place to go for help for experiences of violence (aPR = 1.2). Prevalence ratios of measures related to HIV testing and infection status, HIV risk factors, sexual risk behaviors, and HIV treatment and prevention services did not differ by disability status.

**TABLE 2:** Selected characteristics among adolescent girls and young women aged 13–24 years, by functional disability status (N = 6,318) — Violence Against Children and Youth Survey, Eswatini, 2022

Characteristic	No., unweighted	Weighted % (95% CI)			Chi-square p-value^†^
		Total	With functional disability*	Without functional disability	
**Age group, yrs**					
13–17	3,127	**44.4 (42.7–46.1)**	46.0 (42.2–49.7)	44.2 (42.3–46.0)	0.39
18–24	3,191	**55.6 (53.9–57.3)**	54.0 (50.3–57.8)	55.8 (54.0–57.7)	0.39
**Education^§^**					
Primary school or less	1,183	**17.6 (16.3–19.0)**	19.5 (15.9–23.1)	17.3 (16.0–18.7)	0.23
At least some secondary school	5,132	**82.4 (81.0–83.7)**	80.5 (76.9–84.1)	82.7 (81.3–84.0)	0.23
**Experienced food insecurity^¶^**	3,656	**60.7 (58.5–62.9)**	67.0 (62.7–71.3)	59.7 (57.3–62.0)	0.002
Orphan**	1,663	**28.0 (26.5–29.5)**	28.2 (24.4–32.0)	28.0 (26.4–29.5)	0.90
**Ever been married or lived with someone as if married**	357	**6.0 (5.2–6.8)**	5.6 (3.6–7.5)	6.1 (5.2–7.0)	0.65
**Residence^††^**					
Urban	939	**14.5 (12.3–16.7)**	14.6 (9.1–20.2)	14.5 (12.4–16.7)	0.98
Rural	5,379	**85.5 (83.3–87.7)**	85.4 (79.8–90.9)	85.5 (83.3–87.6)	0.98

* Self-reported, “some difficulty,” “a lot of difficulty,” or “cannot do at all” in one or more functional disability domains (vision, cognition, mobility, self-care, independent living, or communication). Because the ability to hear an interviewer is required for participation in an interviewer-administered survey, the Washington Group Short Set on Functioning question on hearing was not included in the Eswatini Violence Against Children and Youth Survey.

^†^ Rao-Scott chi-square test comparing sociodemographic characteristics by disability status; p-values <0.05 indicate statistical significance.

^§^ Highest level of schooling completed.

^¶^ Household did not have enough money for food.

** Lost one or both parents before age 18 years.

^††^ Lives in an urban or rural area.

**TABLE 3:** Prevalence of experiences of violence, HIV testing and infection status, HIV risk factors and sexual risk behaviors, and use of HIV prevention methods and treatment among adolescent girls and young women aged 13–24 years,* by functional disability status and prevalence ratios comparing prevalence among adolescent girls and young women with and without a functional disability — Violence Against Children and Youth Survey, Eswatini, 2022

Characteristic	Functional disability status				PR (95% CI)	p-value^§^	aPR^¶^ (95% CI)	p-value^§^
	With functional disability^†^		Without functional disability					
	No., unweighted	Weighted % (95% CI)	No., unweighted	Weighted % (95% CI)				
**Experience of violence**								
Lifetime experience of sexual violence	115	14.4 (11.0–17.8)	372	7.1 (6.0–8.1)	2.0 (1.5–2.6)	<0.001	2.0 (1.5–2.6)	<0.001
Lifetime experience of physical violence	154	16.5 (13.5–19.6)	507	9.5 (8.1–10.8)	1.7 (1.3–2.1)	<0.001	1.7 (1.3–2.0)	<0.001
Lifetime experience of emotional violence	298	35.9 (30.7–41.2)	866	15.5 (13.7–17.3)	2.3 (1.9–2.7)	<0.001	2.2 (1.8–2.6)	<0.001
Knew of a place to seek help for experiences of violence	577	67.9 (63.3–72.6)	3,293	59.9 (57.1–62.7)	1.1 (1.0–1.2)	0.004	1.2 (1.1–1.3)	<0.001
Knew of a place to seek help for experiences of sexual violence**	71	58.4 (48.1–68.7)	189	47.9 (41.9–53.8)	1.2 (1.0–1.5)	0.09	1.2 (1.0–1.5)	0.07
Sought professional services for any experience of sexual violence**	35	27.2 (16.1–38.3)	96	25.2 (19.9–30.5)	1.1 (0.6–1.6)	0.75	1.0 (0.5–1.5)	0.97
Received professional services for any experience of sexual violence**	29	24.1 (13.3–34.8)	82	22.8 (17.6–28.0)	1.1 (0.5–1.6)	0.83	1.0 (0.5–1.5)	0.96
Knew of a place to seek help for physical violence^††^	104	68.0 (59.6–76.4)	350	67.9 (61.8–74.1)	1.0 (0.9–1.1)	0.99	1.0 (0.9–1.1)	0.81
Sought professional services for any experience of physical violence^††^	52	32.0 (22.2–41.7)	171	31.3 (25.8–36.7)	1.0 (0.7–1.4)	0.91	1.1 (0.7–1.4)	0.79
Received professional services for any experience of physical violence^††^	32	19.6 (12.5–26.6)	103	17.7 (13.8–21.7)	1.1 (0.6–1.6)	0.65	1.2 (0.7–1.7)	0.47
**HIV testing and infection status**								
Ever tested for HIV infection	657	78.9 (75.5–82.3)	4,203	78.5 (76.9–80.1)	1.0 (1.0–1.1)	0.83	1.0 (1.0–1.0)	0.75
Received positive HIV test result^§§^	69	9.3^¶¶^ (6.5–12.1)	347	6.2*** (5.4–7.0)	1.5 (1.0–2.0)	0.04	1.3 (0.9–1.7)	0.17
Knew HIV infection status^†††^	61	80.6 (62.3–99.0)	314	90.4 (86.9–93.8)	0.9 (0.7–1.1)	0.30	1.0 (0.9–1.1)	0.70
**HIV risk factors and sexual risk behaviors**								
Lifetime experience of transactional sex^§§§^	30	5.4 (3.3–7.6)	168	6.9 (5.5–8.4)	0.8 (0.4–1.1)	0.23	0.8 (0.5–1.2)	0.38
Ever had symptoms or a diagnosis of an STI^§§§^	64	15.5 (10.8–20.2)	261	11.6 (9.6–13.7)	1.3 (0.9–1.8)	0.16	1.3 (0.9–1.8)	0.15
Experienced forced sexual initiation^§§§^	76	17.1 (12.2–21.9)	367	16.6 (14.2–19.0)	1.0 (0.7–1.4)	0.86	1.1 (0.8–1.4)	0.59
Early sexual debut^¶¶¶^	37	8.6 (5.3–11.9)	227	9.4 (7.8–11.0)	0.9 (0.5–1.3)	0.67	1.0 (0.6–1.3)	0.89
Multiple sexual partners (two or more partners during previous 12 months)****	30	7.4 (4.6–10.3)	155	7.5 (6.1–9.0)	1.0 (0.6–1.4)	0.95	1.0 (0.5–1.4)	0.92
Sex partner ≥5 years older****	80	22.1 (16.2–27.9)	566	26.6 (24.3–29.0)	0.8 (0.6–1.1)	0.13	0.9 (0.7–1.1)	0.31
Partner ever refused to wear a condom****	49	14.5 (8.4–20.6)	367	16.7 (14.5–18.9)	0.9 (0.5–1.2)	0.45	0.8 (0.5–1.0)	0.10
Infrequent condom use during previous 12 months****	159	44.4 (37.4–51.4)	920	45.9 (43.0–48.9)	1.0 (0.8–1.1)	0.70	0.9 (0.7–1.1)	0.39
HIV infection status of sex partner is positive or unknown****	89	24.9 (19.4–30.3)	551	26.9 (24.5–29.2)	0.9 (0.7–1.1)	0.47	0.9 (0.8–1.1)	0.59
Fear of experiencing violence from disclosure of HIV status if HIV test result is positive^††††^	56	23.3 (15.1–31.4)	216	15.5 (12.5–18.4)	1.5 (0.9–2.1)	0.09	1.5 (0.9–2.1)	0.12
**HIV prevention methods and treatment**								
Ever heard of PEP^§§§§^	294	36.9 (32.1–41.7)	2,235	41.0 (38.4–43.5)	0.9 (0.8–1.0)	0.10	0.9 (0.8–1.0)	0.08
Ever heard of PrEP^¶¶¶¶^	387	48.8 (44.1–53.5)	2,746	51.3 (48.8–53.8)	1.0 (0.9–1.0)	0.29	0.9 (0.8–1.1)	0.34
Ever taken PEP*****	29	9.4 (5.3–13.6)	240	11.2 (9.3–13.0)	0.8 (0.4–1.2)	0.43	0.9 (0.5–1.3)	0.50
Ever taken PrEP^†††††^	32	12.6 (7.4–17.8)	288	17.7 (14.8–20.5)	0.7 (0.4–1.0)	0.06	0.7 (0.4–1.0)	0.05
On antiretroviral therapy^§§§§§^	54	90.5 (81.2–99.7)	291	93.4 (90.2–96.7)	1.0 (0.9–1.1)	0.54	1.0 (1.0–1.0)	0.83
Viral load suppression^¶¶¶¶¶^	25	53.5 (35.1–71.9)	152	57.7 (50.3–65.0)	0.9 (0.6–1.3)	0.67	0.9 (0.7–1.2)	0.69

**Abbreviations:** aPR = adjusted prevalence ratio; PEP = postexposure prophylaxis; PR = prevalence ratio; PrEP = pre-exposure prophylaxis; STI = sexually transmitted infection; VACS = Violence Against Children and Youth Survey.

* Because of the complex skip patterns used in the VACS, indicator denominators might differ.

^†^ Self-reported, “some difficulty,” “a lot of difficulty,” or “cannot do at all” to one or more functional disability domains (vision, cognition, mobility, self-care, independent living, or communication). Because the ability to hear an interviewer is required for participation in an interviewer-administered survey, the Washington Group Short Set on Functioning question on hearing was not included in the Eswatini VACS.

^§^ Estimates were considered statically significant if Bonferroni-corrected p-values <0.0017.

^¶^ Adjusted for age, education, food insecurity, orphan status, marital status, and residence.

** Among adolescent girls and young women who experienced sexual violence.

^††^ Among adolescent girls and young women who experienced physical violence.

^§§^ Includes those who self-reported a previous positive HIV test result as well as those who received a positive rapid HIV test result at the time of the VACS.

^¶¶^ 7.5% self-report receiving a previous positive HIV test result, and 1.8% had a positive rapid HIV test result at the time of the VACS interview.

*** 5.6% self-report receiving a previous positive HIV test result, and 0.6% had a positive rapid HIV test result at the time of the VACS interview.

^†††^ Among adolescent girls and young women who have received a positive HIV test result.

^§§§^ Among adolescent girls and young women who ever had sex.

^¶¶¶^ Among adolescent girls and young women aged ≥16 years who ever had sex.

**** Among adolescent girls and young women who had sex during the past 12 months.

^††††^ Among adolescent girls and young women who had sex during the past 12 months and have received a negative HIV test result.

^§§§§^ In the questionnaire, PEP is described as follows: “When a person who is HIV-negative takes HIV medicine after a single exposure (such as an unwanted or forced sex experience) to reduce their chances of getting HIV, this is called postexposure prophylaxis, or PEP.”

^¶¶¶¶^ In the questionnaire PrEP is described as follows: “’PREP’ or pre-exposure prophylaxis, involves taking HIV medicine to reduce the chance of getting HIV.”

***** Among adolescent girls and young women who have ever heard of PEP.

^†††††^ Among adolescent girls and young women aged ≥16 years who ever had sex and had heard of PrEP.

^§§§§§^ Among adolescent girls and young women who knew they were living with HIV infection.

^¶¶¶¶¶^ Among adolescent girls and young women who are on antiretroviral treatment and have taken a viral load test.

## Discussion

In Eswatini in 2022, 14% of adolescent girls and young women aged 13–24 years had a self-reported functional disability. Those with a functional disability had a higher lifetime prevalence of experiencing sexual, physical, and emotional violence compared with those without a disability. These findings are consistent with previous studies that found a relationship between disability and experiencing violence (*3**,**5*). Adolescent girls and young women with versus without functional disabilities were also more likely to experience food insecurity, an economic vulnerability that might contribute to increased risk for experiencing violence (*7*). In addition, adolescent girls and young women with a functional disability were more likely to know where to go to seek help services for experiencing violence, potentially because of their increased likelihood of experiencing violence or engagement with health systems where violence referral services might be shared or co-located. However, the extent to which these services are accessible and disability-inclusive is unknown. An analysis of the 2018 Lesotho VACS identified relationships between disability and HIV and risk behaviors (*5*). However, in the current study, disability was not associated with HIV infection status or risk behaviors after adjusting for age, education, food insecurity, orphan status, marital status, and residence. Further investigation is needed to better understand how these factors might modify the relationship between disability and HIV infection status.

Increased prevalences of experiencing sexual, physical, and emotional violence among adolescent girls and young women with functional disabilities highlights the need for accessible and disability-inclusive prevention programming and services to promote continued progress in addressing violence in Eswatini. Preventing experiences of violence among adolescent girls and young women with disabilities aligns with efforts set forth by the Eswatini government (*8**,**9*) to advance the inclusion of persons with disabilities in violence prevention programs and services and ending violence, stigma, and discrimination.

## Limitations

The findings in this report are subject to at least seven limitations. First, self-reported data might be subject to recall, social desirability, or other biases. Second, because VACS are cross-sectional surveys, results cannot be interpreted as being causal or directional. Third, the Bonferroni correction is conservative, and some significant findings might have been missed using the Bonferroni-corrected significance level (e.g., the relationship between disability and HIV infection status). Fourth, including “some difficulty” in the disability categorization might bias results toward the null, because adolescent girls and young women with lesser degrees of functional limitations were included as having a disability. Fifth, because of small response numbers, it was not possible to assess differences in outcomes by disability type or degree of functional limitation, which would be helpful for focusing interventions within a heterogeneous population. Sixth, exclusion of persons with severe disabilities or challenges in understanding or responding to questions might have limited the inclusion of persons with a high degree of functional limitations. Therefore, disability prevalence is an underestimate and results might not be generalizable to that population of adolescent girls and young women. Finally, since hearing disability was not assessed in the Eswatini VACS, adolescent girls and young women with only this disability type would have been excluded when assessing disability.

## Implications for Public Health Practice

Understanding risk factors for experiencing violence by adolescent girls and young women with disabilities might help guide development and implementation of tailored violence prevention programs and services. Prioritizing accessible and disability-inclusive violence prevention programs and service delivery might help reduce experiences of violence among adolescent girls and young women with disabilities (*5*). In addition, violence prevention partners collaborating with disability-led and disability-serving organizations and directly with adolescent girls and young women with disabilities to plan and implement programs and services that are disability-inclusive could help ensure that adolescent girls and young women with disabilities are aware of and can access these resources.

## Footnotes

* Independent living is not included in the WG-SS. A modified version (for country-specific daily errands) of the American Community Survey question on independent living was included in the Eswatini VACS. https://www.census.gov/topics/health/disability/guidance/data-collection-acs.html

† Disability prevalence can be estimated at different cutpoints using WG-SS. The generally recommended threshold for comparing disability prevalence across countries is “a lot of difficulty” or “cannot do at all” for any domain. Under this threshold, the prevalence of functional disability was low (1.7%), resulting in small sample size to allow the comparison of disability status by the outcomes of interest for this analysis.

§ An SAS macro was used to calculate weighted and unweighted prevalence ratios using logistic regression. Sensitivity analyses were run using the SAS (version 9.4; SAS Institute) macro proc genmod to calculate unadjusted prevalence ratios and results were consistent.

¶ Household did not have enough money for food.

** Lost one or both parents before age 18 years.

†† Lives in an urban or rural area.

§§ 45 C.F.R. part 46, 21 C.F.R. part 56; 42 U.S.C. Sect. 241(d); 5 U.S.C. Sect. 552a; 44 U.S.C. Sect. 3501 et seq.
